# Contributions of Inflammatory Processes to the Development of the Early Stages of Diabetic Retinopathy

**DOI:** 10.1155/2007/95103

**Published:** 2007-10-01

**Authors:** Timothy S. Kern

**Affiliations:** ^1^Department of Medicine, Case Western Reserve University, Cleveland, OH 44106-5029, USA; ^2^Department of Ophthalmology and Visual Sciences, Case Western Reserve University, Cleveland, OH 44106-5068, USA; ^3^Cleveland VA Medical Center, 10701 East Boulevard, Cleveland, OH 44106, USA

## Abstract

Diabetes causes metabolic and physiologic abnormalities in the retina, and these changes suggest a role for inflammation in the development of diabetic retinopathy. These changes include upregulation of iNOS, COX-2, ICAM-1, caspase 1, VEGF, and NF-κB, increased production of nitric oxide, prostaglandin E2, IL-1β, and cytokines, as well as increased permeability and leukostasis. Using selective pharmacologic inhibitors or genetically modified animals, an increasing number of therapeutic approaches have been identified that significantly inhibit development of at least the early stages of diabetic retinopathy, especially occlusion and degeneration of retinal capillaries. A common feature of a number of these therapies is that they inhibit production of inflammatory mediators. The concept that localized inflammatory processes play a role in the development of diabetic retinopathy is relatively new, but evidence that supports the hypothesis is accumulating rapidly. This new hypothesis offers new insight into the pathogenesis of diabetic retinopathy, and offers novel targets to inhibit the ocular disease.

## 1. INTRODUCTION

Diabetic retinopathy classically has been regarded as a disease of the retinal microvasculature, and the natural history of the disease has been divided into an early, nonproliferative (or background) stage, and a later, proliferative stage. It is becoming appreciated also that cells of the neuroretina also are affected in diabetes. A number of metabolic or molecular abnormalities that are characteristic of inflammation have been detected in retinas of diabetic animals or patients, or in retinal cells exposed to elevated concentrations of glucose. In the following sections, we will review studies implicating inflammation in the pathogenesis of the early stages of diabetic retinopathy. This review will focus primarily on in vivo studies.

## 2. HISTOPATHOLOGY OF EARLY STAGES OF DIABETIC RETINOPATHY

Histologically, vascular lesions in the early stages of diabetic retinopathy in man and animals are characterized by the presence of saccular capillary microaneurysms, pericyte-deficient capillaries, and obliterated and degenerate capillaries. These degenerate capillaries are not perfused, and so increases in their frequency represent reductions in retinal perfusion.

**Figure 1:**
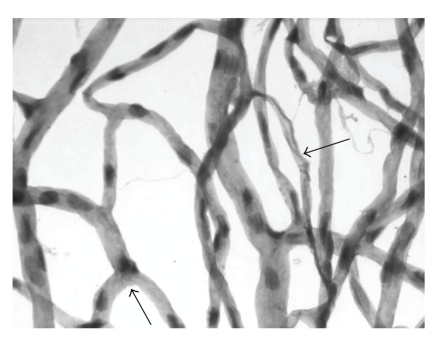
Capillary degeneration in a rat diabetic for 10 months. Large arrow: acellular (degenerate) capillary; small arrow: pericyte ghost.

Capillary occlusion and degeneration initially occurs in single, isolated capillaries, and has no clinical importance when only few capillaries have become nonperfused. As more and more capillaries become occluded, however, retinal perfusion likely decreases, at least locally. Mechanisms believed to contribute to the degeneration of retinal capillaries in diabetes include (1) occlusion of the vascular lumen by white blood cells or platelets, (2) death of capillary cells secondary to biochemical abnormalities within the vascular cells themselves, or (3) capillary cell death secondary to products generated by other nearby cells (such as neurons or glia). All species studied to date have been found to show degeneration of retinal capillaries (Figure 1) as well as death of pericytes and endothelial cells, but microaneurysms are not commonly found in rodent models of diabetic retinopathy.

Diabetes also results in damage to nonvascular cells of the retina. Loss of ganglion cells has been detected in diabetic rats [1–13] and humans [4], but results are controversial in mice [8, 11, 14, 15]. The neurodegeneration in diabetic rats has been detected as early as one month of diabetes [4], thus preceding (and possibly contributing to) the development of the vascular cell changes [4]. The possible role of neurodegeneration in diabetes-induced capillary degeneration has yet to be conclusively demonstrated, but a report that Nepafenac (a COX inhibitor) inhibited diabetes-induced degeneration of retinal capillaries while having no effect on the loss of retinal ganglion cells suggests that the two degenerative events need not be causally linked (16).

Glia and other retinal cells also undergo changes in diabetes in some species. In diabetic rats and humans (but apparently not mice [8]), these cells changed from a quiescent to an injury-associated phenotype with high levels of expressed glial fibrillary acidic protein (GFAP)—a hallmark of glial cell activation [3, 5, 8, 14, 16–20]. Müller glial cells in diabetic rats showed evidence of cell death in some [3, 10], but not all [19], studies. Horizontal cells, amacrine cells, and photoreceptors also have been reported to undergo degeneration in diabetic rats [7, 9], but these changes are not known to be characteristics of retinal changes seen in diabetic patients so their significance remains to be learned. Diabetes-induced changes in retinal function [21–26] are consistent with diabetes causing metabolic alterations in the neural retina.

## 3. INFLAMMATION

Inflammation is a nonspecific response to injury that includes a variety of functional and molecular mediators, including recruitment and activation of leukocytes. Inflammation typically has beneficial effects on an acute basis, but can have undesirable effects if persisting chronically. The increased expression of many inflammatory proteins is regulated at the level of gene transcription through the activation of proinflammatory transcription factors, including NF-κB. These proinflammatory transcription factors are activated and play a critical role in amplifying and perpetuating the inflammatory process. Transcription factors associated with production of proinflammatory mediators include nuclear factor kappa B (NF-κB), activator protein 1 (AP-1), specificity protein 1 (Sp1), peroxisome proliferator-activated receptors (PPARs) and other members of the nuclear receptor superfamily [27–30]. Proinflammatory proteins (including COX-2, interleukin-1, tumor necrosis factor alpha) can contribute to cell damage and death in tissues including brain and retina [31–34], at least in part via activation of NF-κB [32].

## 4. ROLE OF INFLAMMATION IN THE EARLY STAGES OF DIABETIC RETINOPATHY: ANIMAL STUDIES

Many of the molecular and functional changes that are characteristics of inflammation (summarized below) have been detected in retinas from diabetic animals or humans, and in retinal cells cultured in elevated concentrations of glucose. Although many animal species have been studied as possible models of diabetic retinopathy, most of the studies linking inflammatory processes to the development of diabetic retinopathy have been conducted to date in rats and mice, and have focused on insulin-deficient models (type 1 diabetes).

### 4.1. Leukostasis and platelet activation

**Figure 2:**
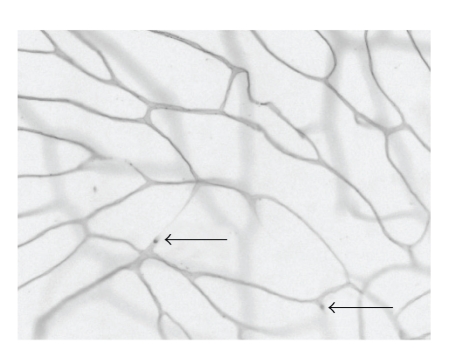
Adherence of white blood cells to the wall of retinal blood vessels (leukostasis). The vasculature of anesthetized animals was perfused with fluorescein-coupled concanavalin A lectin, resulting in stain of all vessel walls and more intense stain of the white blood cells. Occasionally, staining of a capillary was arrested where white blood cells were trapped in the vessel (arrow), suggesting that the blood cell might have occluded the vessel.

Attraction and adhesion of leukocytes to the vascular wall are important components of inflammatory processes. This leukostasis has been found to be significantly increased in retinas of diabetic animals [35–47], and might contribute to the capillary nonperfusion in diabetic retinopathy. Leukocyte stiffness has been reported to be increased in diabetes (decreased filterability) and to contribute to the development of capillary nonperfusion in retinal vessels [36, 48]. A second line of evidence shows that abnormal leukocyte adherence to retinal vessels in diabetes occurs via adhesion molecules. Diabetes increases expression of ICAM-1 in retinas of animals and humans [38, 49] and interaction of this adhesion molecule on retinal endothelia with the CD18 adhesion molecule on monocytes and neutrophils contributes to the diabetes-induced increase in leukostasis within retinal vessels [38]. Leukostasis has been postulated to be a factor in death of retinal endothelial cells in diabetes [40]. Using in situ perfusion methods, evidence consistent with capillary occlusion secondary to leukostasis has been observed in occasional retinal vessels (Figure 2), but it is unclear whether this occurred in vivo or was an artifact caused by the in vitro perfusion. Retinas from diabetic mice lacking ICAM-1 and CD18 are protected from the development of diabetes-induced increase in leukostasis, vascular permeability, and degeneration of retinal capillaries [46], showing these proteins to be important in the development of early stages of diabetic retinopathy. Whether their role in the development of the retinal disease results from capillary occlusion or some other mechanism, however, has not been explored.

A third postulated cause of capillary nonperfusion in diabetes involves platelets. Platelet microthrombi are present in the retinas of diabetic rats and humans, and have been spatially associated with apoptotic endothelial cells [50]. The selective antiplatelet drug (clopidogrel), however, did not prevent neuronal apoptosis, glial reactivity, capillary cell apoptosis, or acellular capillaries in retinas of diabetic rats (51), suggesting that platelets do not initiate the pathology of early diabetic retinopathy.

### 4.2. Increased vascular permeability

Breakdown of the blood-retinal barrier, another early event in the development of diabetic retinopathy, has been attributed to increases in leukostasis, cytokines, and growth factors [40, 51–54]. Increased permeability of the blood retinal barrier is known to occur in patients with diabetes, and this defect contributes to retinal edema and visual impairment in diabetic patients. Controversy remains as to how fast the permeability defect develops in retinas of diabetic animals, with reports ranging from 8 days to more than 6 months after onset of diabetes [41, 55–59]. There has been considerable effort directed towards developing means to assess increased vascular permeability within retinas of animal models, and to identify therapies to inhibit this defect. Therapies that have been found to inhibit the diabetes-induced increase in vascular permeability within the retina include aldose reductase inhibitors, protein kinase C inhibitors, tyrosine kinase inhibitors, aspirin, a COX-2 inhibitor, steroids, VEGF antagonist, TNFα receptor antagonists, and PPAR gamma ligands [41, 47, 56, 57, 60–70].

### 4.3. NF-κB

NF-κB is a widely expressed inducible transcription factor that is an important regulator of many genes involved in mammalian inflammatory and immune responses, proliferation and apoptosis. NF-κB is composed of homodimers and heterodimers, the most abundant and best-studied form in mammalian cells consisting of the p65 and p50 subunits. Activation of NF-κB typically involves the phosphorylation of cytoplasmic IκB by the IκB kinase (IKK) complex, resulting in IκB degradation via the proteosomal system. The degradation of IκB releases the NF-κB heterodimers to translocate to the nucleus where they bind to nuclear DNA, leading to activation of specific subsets of genes. DNA-binding experiments (EMSA) have demonstrated NF-κB to be activated in retinal endothelial cells or pericytes exposed to elevated glucose concentration and in retinas of diabetic rats [71, 72]. Diabetes has been found to cause migration of the p65 subunit into the nucleus of retinal pericytes [73], and of the p50 subunit into nuclei of retinal endothelial cells, pericytes, ganglion cells, and cells of the inner nuclear layer [74].

Evidence in support of an important role of NF-κB in the pathogenesis of early stages of diabetic retinopathy is twofold. First, inhibition of proteins whose expression is regulated by NF-κB (such as iNOS and ICAM) inhibit diabetes-induced degeneration of retinal capillaries (described below). Second, compounds known to inhibit NF-κB likewise inhibit the development of the retinopathy. For example, several different antioxidants which inhibit the development of capillary degeneration and pericyte loss in retinas of diabetic rats [75] also inhibit the diabetes-induced activation of retinal NF-κB (72). Likewise, low-intermediate doses of salicylates (aspirin, sodium salicylate, and sulfasalazine) which inhibited NF-κB activation in retinas of diabetic rats, also inhibited expression of inflammatory mediators like iNOS and ICAM-1, and capillary degeneration and pericyte loss in those animals (75; 77). Aspirin is known to inhibit also production of prostaglandins, but salicylate and sulfasalazine have much less of this activity, suggesting that the common action of these 3 salicylates to inhibit retinopathy in diabetes was not primarily mediated by inhibition of prostaglandins.

### 4.4. iNOS

iNOS expression is regulated at least in part by NF-κB. Interestingly, experimental sympathectomy itself increases gene and protein expression of iNOS in retinas of nondiabetic rats (78), suggesting that loss of sympathetic activity, such as which occurs in diabetes, might contribute to the upregulation of this inflammatory protein in the retina.

In retinas of diabetic animals, increased levels of nitric oxide products (nitrotyrosine, nitrite, nitrate) have been reported [76–78]. Upregulation of iNOS has been found in retinas of experimental diabetic rodents and patients in most studies [33, 55, 76, 78–82]. Diabetes-induced alterations in expression of other isoforms of nitric oxide synthase also have been reported [83, 84]. A possible role of iNOS in the pathogenesis of diabetic retinopathy is suggested by the studies of aminoguanidine. Aminoguanidine is a relatively selective inhibitor of iNOS [85–88], and has been found to inhibit the diabetes-induced increase nitric oxide production and iNOS expression in retina [78].

Aminoguanidine also has been found to inhibit the development of the microvascular lesions of diabetic retinopathy in diabetic dogs [89], rats [90–92], and mice (Kern, unpublished data). Nevertheless, aminoguanidine also has other effects [93–100], so this therapy does not absolutely prove a role of iNOS in the pathogenesis of the retinopathy.

The role of iNOS in the development of the early stages of diabetic retinopathy recently has been investigated directly using mice genetically deficient in iNOS [101]. In that study, wildtype diabetic mice developed the expected degeneration of retinal capillaries, as well as increase in leukostasis and superoxide generation. In contrast, diabetic mice deficient in iNOS did not develop these structural or functional abnormalities.

eNOS expression also has been reported to be elevated in the retinas in the diabetic rats, and it has been suggested that eNOS might play a role in the development of diabetes-induced leukostasis and/or retinopathy [41, 56, 83]. This possibility has not been experimentally addressed due, in part, to the hypertension that results in the absence of eNOS, as well as a lack of specific inhibitors of the enzyme.

### 4.5. Cyclooxygenases

COX-2 expression is regulated at least in part by NF-κB. In retinas of diabetic animals, induction of COX-2 as well as increased production of prostaglandins has been reported [33, 67, 102–104]. Ayalasomayajula and coworkers [104] have shown that PGE_2_ production by retinas from diabetic rats was significantly inhibited by celecoxib (a selective COX2 inhibitor), but not by a COX-1 inhibitor, suggesting that COX-2 is primarily responsible for the diabetes-induced increase in retinal production of PGE_2_ in diabetic rats. Inhibition of COX-2 has been reported to inhibit the diabetes-induced upregulation of retinal prostaglandins and VEGF [67], the increase in retinal vessel permeability and leukostasis [41], and the death of retinal endothelial cells cultured in diabetic-like concentrations of glucose [33]. The COX-2 inhibitor, Meloxicam, also reduced eNOS levels, inhibited NF-κB activation in the diabetic retina, and modestly, but significantly, reduced TNFα levels in the retina [41]. Its effect on histologic lesions of diabetic retinopathy was not studied.

Less selective COX inhibitors have inhibited the development of the retinopathy in diabetic dogs and rodents [74, 89], as well as the increase in vascular permeability in diabetic rodents [41]. Nepafenac is an inhibitor of cyclooxygenases that can be applied in eye drops. It was found to inhibit diabetes-induced prostaglandin production and leukocyte adhesion in retinal vessels of diabetic rats, and the diabetes-induced increase in the number of TUNEL-positive capillary cells, acellular capillaries, and pericyte ghosts in the retina [21].

### 4.6. ICAM-1

White blood cells bind to ICAM-1 on the surface of endothelial cells as a component of a multistep process leading to adherence of the white blood cell to the endothelial wall [38]. This leukostasis is known to be increased in retinal blood vessels in diabetes [21, 38, 40–42, 44, 46, 56, 105, 106], and this process is mediated via ICAM-1 [38]. ICAM-1 is upregulated by several stimuli, including VEGF, PARP activation, oxidative stress, and dylipidemia [72, 107–109], at least in part by NF-κB.

Genetically modified C57B1/6J mice recently have been used to explore the roles of ICAM-1 and its ligand on white blood cells (CD18) in the pathogenesis of diabetes-induced retinal vascular disease [46]. Mice deficient in the genes for these proteins and their wildtype controls were made diabetic or experimentally galactosemic. After durations of up to 11 months (diabetes) or 22 months (galactosemia), wildtype diabetic or galactosemic animals developed capillary degeneration and pericyte loss as well as associated abnormalities including leukostasis, increased capillary permeability and capillary basement membrane thickening. In contrast, CD18^−/−^and ICAM-1^−/−^mice developed significantly fewer of each of these abnormalities, thus providing evidence that these inflammatory proteins play an important role in the pathogenesis of the retinopathy.

### 4.7. VEGF

VEGF is a proinflammatory molecule that plays a well-recognized role in neovascularizaton and in increased permeability. VEGF expression is regulated largely by hypoxia, but it also accumulates in the retina early in diabetes, before any retinal hypoxia is yet apparent [110–112]. It is produced by multiple cell types in the retina in diabetes, including ganglion cells, Mueller cells, and pericytes. Repeated injections of high concentrations of VEGF in the eyes of nondiabetic monkeys result in retinal changes which in some ways resemble those in the early stages of diabetic retinopathy, including vascular tortuosity and microaneurysms [113, 114]. Clinical trials using anti-VEGF therapies are showing promising results against advanced stages of diabetic retinopathy [115–121].

### 4.8. IL-1β and caspase-1

Levels of the proinflammatory cytokine, IL-1β, are known to be increased in retinas from diabetic rats [34, 122, 123]. Intravitreal injection of IL-1β or exposure of retinal endothelial cells to the cytokine in vitro was shown to be capable of causing degeneration of retinal capillary endothelial cells [32], but the relevance of these findings to capillary degeneration in vivo is not clear because the levels of IL-1β likely were pharmacologically high. The role of IL-1β in the pathogenesis of diabetic retinopathy recently has been more directly studied using diabetic mice in whom the enzyme responsible for IL-1β production was inhibited or in whom the IL-1β receptor was deleted. IL-1β is the predominant product of caspase-1, and the biological activity of IL-1β is mediated by binding to the cell surface receptor, IL-1R1. Activity of caspase-1 is increased in retinas of diabetic mice, galactose-fed mice, and diabetic humans, and in retinal Müller cells incubated in elevated glucose concentration [124]. Inhibition of caspase-1 using minocycline inhibited the diabetes-induced increase in IL-1β and decreased degeneration of retinal capillaries in those animals [34]. Likewise, inhibition of IL-1β signaling using IL-1β receptor knock-out mice protected the animals from diabetes-induced retinal pathology at 7 months duration of diabetes [34]. The results indicate that activation of caspase-1 and subsequent production of IL-1β play an important role in the development of diabetes-induced retinal pathology. One known action of IL-1β is to activate NF-κB.

### 4.9. TNFα and other cytokines

Retinal levels of TNFα are significantly greater than normal in diabetic rats [41, 125]. Eternacept is a soluble TNFα receptor that acts as competitive inhibitor to block effects of TNFα binding to cells. Eternacept reduced leukocyte adherence in retinal blood vessels of rats diabetic for 1 week compared to control [41]. Eternacept did not reduce retinal VEGF levels, but it inhibited blood-retinal barrier breakdown and NF-κB activation in the diabetic retina. No effects of the therapy on histologic lesions of the retinopathy were evaluated in diabetic animals, but mice genetically deficient in TNF were reported in an abstract to be protected from galactose-induced retinopathy [126]. Epiretinal membranes obtained by vitrectomy, as well as cultured Muller glial cells stimulated with glycated albumin or high glucose, showed increased expression of monocyte chemotactic protein-1 mRNA and protein [127]. These studies suggested that monocyte chemotactic protein-1, under the regulation of NF-κB, is a component of the diabetes-induced inflammation in the retina.

### 4.10. Fas

Fas levels are increased in retinas of diabetic rats [41, 126, 128]. Blocking FasL in vivo has been shown to prevent endothelial cell damage, vascular leakage, and platelet accumulation in diabetes, suggesting that the Fas/FasL system might contribute to the diabetes-induced damage that contributes to the development of the retinopathy [128], but its role in the development of retinal histopathology has not been assessed.

### 4.11. Complement

Deposition of C5b-9, the terminal product of complement activation, has been observed within retinal blood vessels of diabetic rats and humans [129]. Endogenous inhibitors of complement activation, including CD55, CD59, and DAF, have been observed to have subnormal expression or impaired function as a result of nonenzymatic glycation [130–132]. Whether or not inhibition of the complement system can inhibit the development of lesions characteristic of the retinopathy remains to be learned.

### 4.12. Angiopoietin-1

Angiopoietin-1 has been found to have anti-inflammatory actions, including inhibition of vascular permeability and adhesionprotein expression [133]. When administered intravitreally to diabetic rats, angiopoietin-1 normalized blood-retinalbarrier function, leukostasis and endothelial injury, and inhibited upregulation of retinal VEGF and ICAM-1 mRNA and protein [56].

## 5. SEVERAL THERAPIES THAT INHIBIT RETINOPATHY ARE KNOWN TO INHIBIT NF-κB

### 5.1. PARP

**Figure 3:**
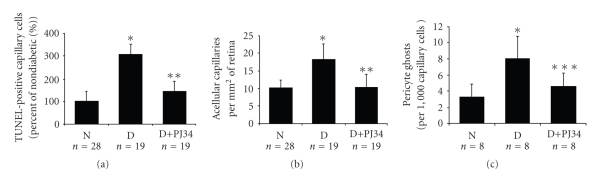
PARP inhibitor inhibits retinal capillary cell death and development of lesions of diabetic retinopathy ((a) TUNEL-positive cells, (b) acellular capillaries, and (c) pericyte ghosts). (N: nondiabetic rats; D: diabetic rats; D+PJ-34: diabetic rats treated with PJ-34. ^*^ P<.005 compared to nondiabetic control, ^**^ P<.0001 compared to diabetic control, and ^***^ P<.02 compared to diabetic control.) Reprinted by permission from *Diabetes* Vol. 53; pp. 2960—2967; 2004*©*The American Diabetes Association.

 Administration of a potent PARP inhibitor (PJ34) for nine months to diabetic rats significantly inhibited the diabetes-induced death of retinal microvascular cells and the development of early lesions of diabetic retinopathy, including capillary degeneration [72] (Figure 3). Evidence suggests that the inhibitor exerts this beneficial effect at least in part by regulating activation of the transcription factor, NF-κB, and in particular, the p50 subunit of NF-κB. In bovine retinal endothelial cells, PARP interacts directly with subunits of NF-κB, and inhibition of PARP activity blocked the hyperglycemia-induced increase in NF-κB and proinflammatory gene products [72].

### 5.2. Antioxidants

Antioxidants have been found to inhibit the development of inflammatory changes in retinas of diabetic animals, including activation of NF-κB, leukostasis, and increased expression of iNOS [71, 134]. Consistent with this, antioxidants have been found to partially, but significantly, inhibit the development of acellular capillaries and pericyte ghosts in diabetic rats. Mixtures of α-tocopherol and ascorbate [75], of α-tocopherol, ascorbate, Trolox, acetylcysteine and selenium [75], α-tocopherol alone (Kern, unpublished), and lipoic acid [135] have been found to significantly inhibit the development of acellular capillaries in retinas of diabetic rodents. The antioxidant and lipid-lowering agent, nicanartine, significantly inhibited diabetes-induced alterations in the number of retinal capillary endothelial cells and pericytes in rats, but had no effect on the formation of acellular capillaries [136].

### 5.3. Benfotiamine

Benfotiamine is a lipid-soluble thiamine derivative that is known to activate transketolase, and is believed to divert sugar metabolites away from glycolysis [137]. Benfotiamine significantly inhibited several hyperglycemia-induced abnormalities, including activation of NF-κB [137]. In addition, administration of benfotiamine significantly inhibited the development of acellular capillaries in retinas of diabetic rats [137]. Whether or not this beneficial effect of the drug on histopathology of the retina was secondary to regulation of NF-κB has not been investigated.

### 5.4. Advanced glycation endproducts (AGEs) and their receptors

 Binding of AGEs or other related molecules to their extracellular receptors such as RAGE (receptor for advanced glycation endproducts) have a variety of intracellular effects, including activation of the proinflammatory NF-κB and stimulation of leukostasis [138–143]. Pharmacological interventions interrupting RAGE-ligand interaction inhibit diabetes-induced degeneration of retinal capillaries in diabetes [25], but whether or not this is mediated by inhibition of NF-κB has not been explored.

### 5.5. Aldose reductase

Inhibition of the polyol pathway enzyme aldose reductase has been reported to inhibit expression of ICAM-1, VCAM-1, COX-2 expression and leukostasis via inhibition of NF-κB activity and nuclear translocation, and phosphorylation and degradation of Iκ-Bα [144–146]. The role of NF-κB regulation in reported effects of aldose reductase inhibitors on the development of retinopathy is unclear.

### 5.6. Corticosteroids

Corticosteroids are known to exert major anti-inflammatory effects. Intravitreal injection of such steroids has been found to inhibit diabetes-induced alterations in permeability of the retinal vasculature and retinal edema in patients [15–133, 135–156].

## 6. THERAPIES INHIBITING INFLAMMATION AND RETINOPATHY IN MULTIPLE WAYS

### 6.1. Minocycline 

 Minocycline is a second-generation, chemically modified tetracycline [157] that exerts pleiotropic actions including anti-inflammatory effects distinct from its antimicrobial action [158, 159]. Minocycline has neuroprotective qualities in models of cerebral ischemia, traumatic brain injuries, ALS, Huntington's, and Parkinson's disease in mice [160–169]. It has been speculated that its neuroprotective action is mediated by the inhibition of activation of caspase-1 and caspase-3, inhibition of generation of IL-1β, and iNOS [170, 171]. Minocycline also inhibits activation of retinal microglia induced either by lipopolysaccharide or by diabetes, and prevents early caspase-3 activity and neuronal apoptosis in the retina of diabetic rats [123, 172]. Long-term administration of minocycline also significantly inhibited the degeneration of retinal capillaries in diabetic mice and galactose-fed mice [34].

### 6.2. Aspirin and salicylates

Aspirin is known to inhibit production of prostaglandins as a result of cyclo-oxygenase inhibition. Sodium salicylate and sulphasalazine have less of this activity, however, but all of these salicylates were able to inhibit capillary degeneration in retinas of diabetic rats [74], suggesting that their common action to inhibit retinopathy was via inhibition of the NF-κB pathway. Whether this occurs via direct or indirect actions remains to be learned.

### 6.3. Aldose reductase inhibitors

Aldose reductase inhibitors have long been studied for their ability to inhibit aldose reductase under hyperglycemic conditions. The ability of this class of drugs to inhibit diabetic retinopathy has been mixed in animals [8, 173, 174], and unsuccessful in diabetic patients [175, 176]. Recently, aldose reductase inhibitors have been found to have potent anti-inflammatory actions, even in normoglycemia [144–146, 177]. The possibility that reported beneficial effects of aldose reductase inhibitors on diabetic retinopathy were due, instead, to anti-inflammatory actions has not yet been studied.

## 7. ARE DIABETES-INDUCED INFLAMMATORY CHANGES IN THE RETINA INDEPENDENT OF EACH OTHER, OR ARE THEY INTERRELATED?

**Figure 4:**
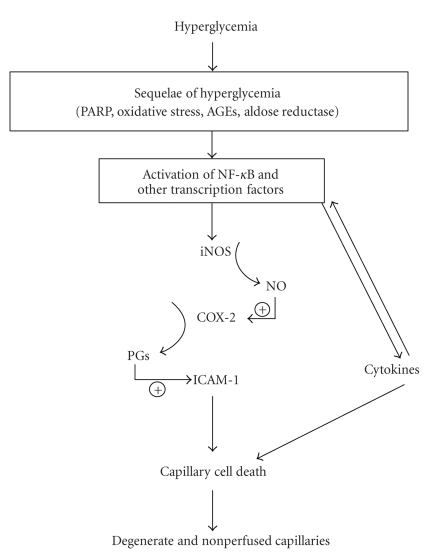
Working hypothesis of the contribution of inflammatory processes in the pathogenesis of capillary degeneration and other lesions of early diabetic retinopathy. The capillary degeneration can be inhibited in diabetic animals at any of several different points along this pathway.

Many of the inflammatory proteins shown above to be involved in the diabetes-induced degeneration of retinal capillaries are known to be regulated by NF-κB. It is conceivable that each of these proteins independently cause the capillary degeneration, but several pieces of evidence suggest that they act in a sequential, hierarchical pathway like that summarized in Figure 4. Evidence using retinal tissue from diabetic animals or incubated in high glucose indicates that (a) PARP regulates activity of NF-κB as well as expression of ICAM-1 [72], (b) inhibition of NF-κB with sulfasalazine inhibits expression of iNOS, ICAM-1, VCAM, COX-2 [74, 148], (c) inhibition of iNOS inhibits the hyperglycemia-induced generation of prostaglandin [33], whereas the opposite reaction (regulation of nitric oxide production by COX-2) was not detected, and (d) inhibition of COX inhibits expression of ICAM-1 and leukostasis [21]. This pathway undoubtedly will become more complicated and interactive as more information becomes available about the role of proinflammatory proteins and transcription factors in the development of diabetic retinopathy. Many cytokines are known to activate NF-κB and other proinflammatory mediators, thus, even now suggesting considerable complexity in the initiation and regulation of this pro-inflammatory “pathway.”

## 8. IS NF-κB THE ONLY REGULATOR OF INFLAMMATORY GENE TRANSCRIPTION IN DIABETIC RETINOPATHY?

Multiple transcription factors have been shown to regulate inflammation, so it seems unlikely that NF-κB is the only regulator of diabetes-induced inflammation in diabetic retinopathy. Retinas from diabetic rats have been reported to have increased expression of another transcription factor, CCAAT/enhancer-binding protein-beta [147], but this was not confirmed [148]. HIF-1α expression in retinas of diabetic NOD mice increased with duration of diabetes, increased immunostaining for HIF-1α being demonstrated in the inner (but not outer) retina [178]. To date, other transcription factors involved in regulation of inflammation seem not to have been studied in vivo in relation to diabetic retinopathy.

## 9. INFLAMMATION IN HUMAN DIABETIC RETINOPATHY

Evidence that inflammatory processes play an important role in the degeneration of retinal capillaries in diabetic patients is less complete than that in animals, but is in many ways consistent with the animal studies. Increases in levels of TNFα, IL -1β , and other inflammatory mediators have been shown in vitreous of diabetic patients [179–184]. Activity of caspase-1, the enzyme responsible for production of IL-1β , is increased in retinas of diabetic humans, and correlates with the distribution of lesions in the retina [185]. Deposition of C5b-9, the terminal product of complement activation, has been observed within retinal blood vessels of diabetic humans [129].

Prospective clinical trials to assess the possible effect of aspirin on diabetic retinopathy in patients have yielded contradictory results. Aspirin treatment resulted in a statistically significant (although weak) inhibition of the mean yearly increase in the number of microaneurysms in the DAMAD trial [186], whereas no beneficial effect was observed on any aspect of retinopathy in the ETDRS trial [187]. The lack of effect of aspirin in the ETDRS is likely attributable, in part, to the greater severity of retinopathy at the onset than in the DAMAD trial or animal studies, and the lower doses of aspirin used. In light of the different conclusions reached in these clinical trials, and positive results achieved in animal studies, it seems prudent to reserve judgement at this time about whether or not aspirin might inhibit diabetic retinopathy in humans.

## 10. CONCLUSIONS

 In composite, numerous defects that develop in retinas as a result of diabetes are consistent with a diabetes-induced inflammatory response in that tissue. These inflammatory changes apparently are important in the pathogenesis of diabetic retinopathy, since inhibition of this inflammatory cascade at any of multiple steps can inhibit the early stages of diabetic retinopathy (notably, degeneration of retinal capillaries) in animals. Findings of diabetes-induced inflammatory changes, generally, in the human eye also, are consistent with the postulate that inflammatory processes contribute to the development of diabetic retinopathy. The evidence in diabetic animals is sufficient to warrant further investigations of the role of inflammation in the development of diabetic retinopathy in patients.

## ABBREVIATIONS

iNOS: Inducible isoform of nitric oxide synthase
COX: Cyclooxygenase
ICAM: Intercellular adhesion molecule
VEGF: Vascular endothelial growth factor
NF-κB: Nuclear factor kappa beta
IL-1β: Interleukin 1beta

TNFα: Tumor necrosis factor alpha
EMSA: Electromobility shift assay
eNOS: Endothelial isoform of nitric oxide synthase
PARP: Poly(ADP-ribose) polymerase
